# Exploring Efficacy of a Serious Game (Tobbstop) for Smoking Cessation During Pregnancy: Randomized Controlled Trial

**DOI:** 10.2196/12835

**Published:** 2019-03-27

**Authors:** Francesc X Marin-Gomez, Rocio Garcia-Moreno Marchán, Anabel Mayos-Fernandez, Gemma Flores-Mateo, Esther Granado-Font, Maria Luisa Barrera Uriarte, Jordi Duch, Cristina Rey-Reñones

**Affiliations:** 1 Servei d'Atenció Primària d'Osona Gerència Territorial de la Catalunya Central Institut Català de la Salut Vic Spain; 2 Unitat de Suport a la Recerca Catalunya Central Institut Universitari d'Investigació en Atenció Primària Jordi Gol Sant Fruitós de Bages Spain; 3 Health Promotion in Rural Areas Research Group Institut Català de la Salut Sant Fruitós de Bages Spain; 4 Digital Care Research Group Universitat de Vic–Universitat Central de Catalunya Centre for Health and Social Care Research Vic Spain; 5 Sexual and Reproductive Health Unit Servei d'Atenció Primària d'Osona Institut Català de la Salut Vic Spain; 6 Grup de Recerca en Tecnologies de la Informació en Atenció Primaria Unitat de Suport a la Recerca Tarragona-Reus Institut Universitari d'Investigació en Atenció Primària Jordi Gol Reus Spain; 7 Unitat d’Anàlisi i Qualitat Xarxa Sanitària i Social Santa Tecla Tarragona Spain; 8 Departament d'Infermeria Facultat d'Infermeria Universitat Rovira i Virgili Tarragona Spain; 9 Centre d'Atenció Primària Horts de Miró (Reus-4) Gerència d'Àmbit d'Atenció Primària Camp de Tarragona Institut Català de la Salut Tarragona Spain; 10 Centre d’Atenció Primària La Granja (Tarragona-2) Gerència d’Àmbit d’Atenció Primària Camp de Tarragona Institut Català de la Salut Torreforta, Tarragona Spain; 11 Departament d'Enginyeria Informàtica i Matemàtiques Universitat Rovira i Virgili Tarragona Spain

**Keywords:** pregnancy, video games, smoking cessation

## Abstract

**Background:**

Tobacco use during pregnancy entails a serious risk to the mother and harmful effects on the development of the child. Europe has the highest tobacco smoking prevalence (19.3%) compared with the 6.8% global mean. Between 20% to 30% of pregnant women used tobacco during pregnancy worldwide. These data emphasize the urgent need for community education and implementation of prevention strategies focused on the risks associated with tobacco use during pregnancy.

**Objective:**

The aim of this study was to investigate the efficacy of an intervention that incorporates a serious game (Tobbstop) to help pregnant smokers quit smoking.

**Methods:**

A two-arm randomized controlled trial enrolled 42 women who visited 2 primary care centers in Catalonia, Spain, between March 2015 and November 2016. All participants were pregnant smokers, above 18 years old, attending consultation with a midwife during the first trimester of pregnancy, and had expressed their desire to stop smoking. Participants were randomized to the intervention (n=21) or control group (n=21). The intervention group was instructed to install the game on their mobile phone or tablet and use it for 3 months. Until delivery, all the participants were assessed on their stage of smoking cessation during their follow-up midwife consultations. The primary outcome was continuous tobacco abstinence until delivery confirmed by the amount of carbon monoxide at each visit, measured with a carboxymeter.

**Results:**

Continuous abstinence until delivery outcome was 57% (12/21) in the intervention group versus 14% (3/21) in the control group (hazard ratio=4.31; 95% CI 1.87-9.97; *P*=.001). The mean of total days without smoking until delivery was higher in the intervention group (mean 139.75, SD 21.76) compared with the control group (mean 33.28, SD 13.27; *P*<.001). In addition, a Kapplan-Meier survival analysis showed that intervention group has a higher abstinence rate compared with the control group (log-rank test, *χ*^2^_1_=13.91; *P*<.001).

**Conclusions:**

Serious game use is associated with an increased likelihood to maintain abstinence during the intervention period if compared with those not using the game. Pregnancy is an ideal opportunity to intervene and control tobacco use among future mothers. On the other hand, serious games are an emerging technology, growing in importance, which are shown to be a good tool to help quitting smoking during pregnancy and also to maintain this abstinent behavior. However, because of the study design limitations, these outcomes should be interpreted with caution. More research, using larger samples and longer follow-up periods, is needed to replicate the findings of this study.

**Trial Registration:**

ClinicalTrials.gov NCT01734421; https://clinicaltrials.gov/ct2/show/NCT01734421 (Archived by WebCite at http://www.webcitation.org/75ISc59pB)

## Introduction

### Background

Mobile health (mHealth) is defined by the World Health Organization as “medical and public health practice supported by mobile devices, such as mobile phones, patient monitoring devices, personal digital assistants (PDAs), and other wireless devices.” In 2017, the estimated number of available mHealth apps increased to approximately 325,000 [1]. Unfortunately, despite the fact that there is a large production of apps that aimed to improve health, we do not know scientifically the impact their use has on the user’s health [2].

Preventive medicine is one of the aims of primary healthcare (PHC). The prevention of smoking habits is one of the most important preventive care practices undertaken in PHC. Smoking is claimed to have caused more than 1 trillion deaths only in the 21st century [3]. Tobacco use during pregnancy constitutes, in addition to a serious risk to the mother, harmful effects on the development of the child, making of pregnancy an ideal opportunity to intervene and control tobacco use among mothers and families.

In Spain, 28.3% of childbearing-aged women smoke on a daily basis. Research reveals that almost 24% of cessations occurred once pregnancy was confirmed [4]. In European countries, the proportion of women who smoke regularly or occasionally during different periods of pregnancy varies from 8.5% in Germany, 12.0% in England, 14.4% in Catalonia (Spain), and 17.1% in France [5]. Previous studies show a high percentage of relapses during postpartum [6,7], and it would seem that under this, low abstention could conceal problems related to the lack of knowledge and education about the harmful effects of smoking during pregnancy.

A systematic review and meta-analysis that included 77 clinical trials and about 29,000 women showed that the interventions carried out during pregnancy were effective in reducing tobacco consumption only when the advice was made in the consultations with other interventions [8]. Digital interventions, particularly those delivered by short message service (SMS) text message, or computer, can be effective for smoking cessation in pregnancy, and digital interventions containing behavior change techniques focused around goals setting, problem solving, and action planning could even be better [9]. Digital games have been evaluated for smoking cessation, and mobile apps are replacing money rewards for virtual goods to help players meet game objectives and incentivize bio-verified abstinence [10].

Anyone who has observed someone absorbed in a mobile device game might have seen that the use of these games provides a very powerful interaction. “For the player, time stops and self-consciousness disappears.” Csikszentmihalyi describes this state as *flow* [11], and it could very well describe what happens when an individual gets involved in one of the interactive games that we can today download easily on our mobile.

### Objectives

The advanced processing capabilities, global reach, and unmatched accessibility of smartphones render them ideal channels for delivering health-related interventions [12]. The complex functionalities enabled in the apps facilitate high user engagement, which is a strong predictor of smoking cessation [13].

Although there is a growing body of evaluated evidence on the efficacy and effectiveness of smartphone-based technologies for smoking cessation, this has not been studied enough in a very sensitive group such as pregnant women, and although most evaluative evidence consists of SMS text messaging–based interventions [14,15] and supporting follow-up apps [16], there is a lack of evidence in relation to serious games.

The best way to create an effective technique is to gain complete understanding of users’ tastes and needs. This information will be key in the development of future mobile apps that respond and are better adapted to user demands, [14]. This study evaluates the effectiveness of a serious game app (Tobbstop) [17] on pregnant women who want to quit smoking, as a way to help understanding user’s trends.

### Tobbstop Trial

The Tobbstop trial was a multicenter randomized clinical trial [18] carried out in Catalonia (Spain) that included the recruitment centers of this study. The general aim of the study was to assess the efficacy of a serious game app for smoking cessation. Smokers were recruited from primary health care centers and were randomized into 2 groups: (1) an intervention group that included access to the Tobbstop serious game and the usual counseling about smoking cessation and (2) a control group that received only the usual smoking cessation counseling. This study analyzed their impact in the subgroup of pregnant women participating.

## Methods

### Design

**Figure 1:**
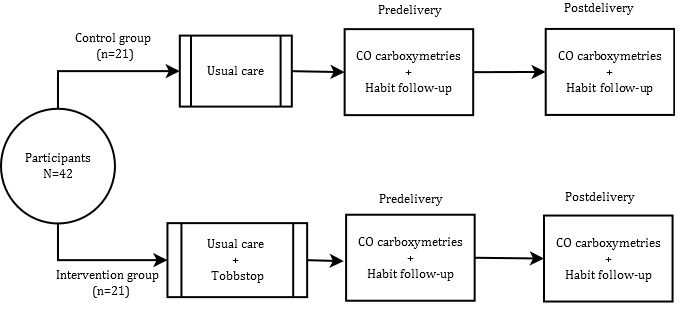
Study design flowchart. CO: carbon monoxide.

The trial was a prospective, randomized, 2-armed controlled pilot study conducted at 2 Sexual and Reproductive Health Units (SRHUs) at PHC centers in Catalonia from March 2015 to October 2016. Study participants were assigned to either an intervention group (n=21) or a control group (n=21) using a block randomization technique (eg, for 1 week, participants were randomized to the intervention group, and the following week, participants were randomized to the control group). Pregnant women in the intervention group started playing Tobbstop 1 week before the day chosen for cessation (D-day), and played until 90 days after D-day [18]. Before the day of cessation, degree of nicotine dependence was measured with the Fagerström test and the motivation to stop smoking was assessed by Richmond test. The testing protocol for the control subjects received standard of care and only differed in that they did not receive the Tobbstop app (use of the game), whereas the assessments remained the same for intervention and control group (Figure 1). The study was conceived as a pilot trial.

**Figure 2:**
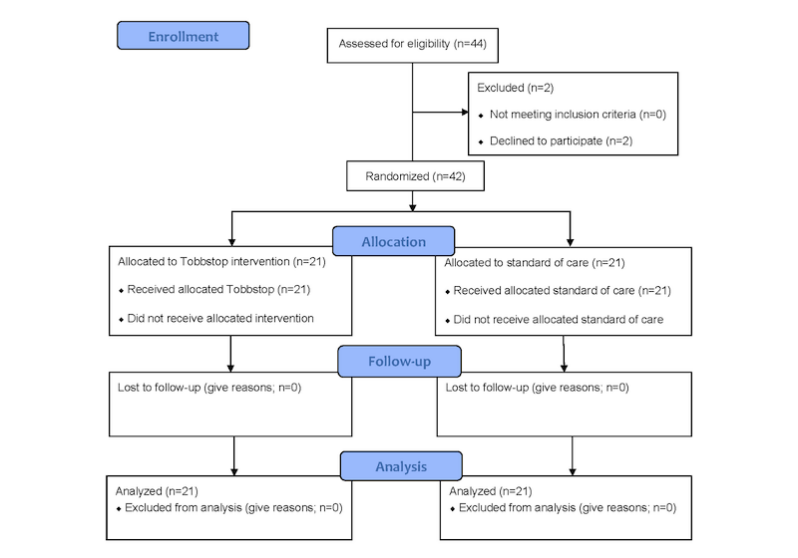
CONSORT flow diagram.

The protocol was in accordance with the Consolidated Standards of Reporting Trials (CONSORT)-EHEALTH checklist and a CONSORT diagram of the proposed study design is shown (Figure 2). A complete description of the main study protocol was published elsewhere [18].

### Participants

A total of 44 pregnant smokers were contacted for inclusion in the study. Finally, we included 42, which were allocated to either the intervention group or the control group. Inclusion criteria were as follows: (1) aged older than 18 years, (2) active smokers, (3) motivation to quit smoking ≥6 points on the Richmond test, and (4) have a mobile device with Android or iPhone operating system. Written informed consent was obtained from all women included in the study.

### Follow-Up Visits

During follow-up visits with the midwife, the woman’s health history was taken note of, and first tips on healthy habits and disease prevention were given as usual. Frequencies of visits throughout pregnancy depended on the individual needs of each woman and were based on their associated risk factors. Despite this, it is recommended that a minimum of 9 prenatal visits should be made for a woman with a normal pregnancy, with the following periodicity: before 10 weeks, at 11 to 13 weeks, 16 to 17 weeks, 20 to 21 weeks, 25 to 26 weeks, 29 to 30 weeks, 34 to 36 weeks, 38 to 40 weeks, and finally, at 41 weeks of gestation.

In our study, the same calendar of follow-up visits was used to monitor the study variables, but the use of Tobbstop was only proposed for the intervention group. In the recruitment visit, we collected data on tobacco consumption: (1) number of cigarettes smoked per day, (2) number of cigarettes smoked before pregnancy, (3) age of onset, and (4) number of previous attempts to quit and presence of smokers in the family environment.

The intervention group accessed Tobbstop from their smartphones and played from 7 days before their smoking cessation day (D-day) to 90 days after.

### The Tobbstop App

A multidisciplinary team of health experts, pedagogues, computer engineers, graphic designers, and video game developers created Tobbstop, a mobile app dedicated to smoking cessation, in 2015 [19]. Its purpose was accompanying the process of quitting for the first 90 days, the most critical days for a possible relapse.

**Figure 3:**
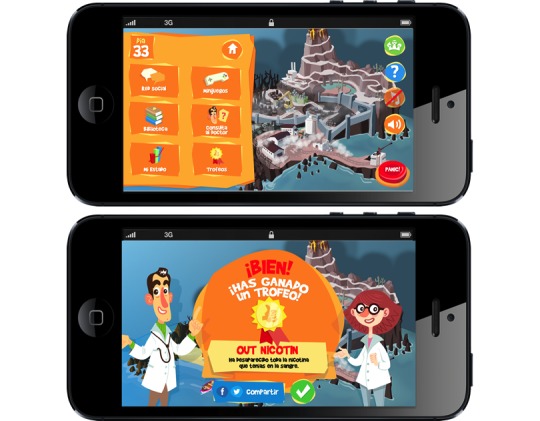
Screenshots of Tobbstop.

The app offers support during the different stages of the smoking cessation process, combining strategies of electronic health, gamification, and mobile learning. It represents the way forward when you stop smoking in the form of a video game. The game is played on an island that is initially polluted, dirty, and contaminated, a metaphor of a smoker's body, and it walks the player through the process of detoxification. The objective of the player is to clean and purify the island while improving it (Figure 3). This is achieved by appropriately using the 4 available tools, which are: (1) access to data source of health education on snuff; (2) access to a social network that allows the player to communicate with other study participants, share experiences and concerns, and provide mutual support [19]; (3) a group of *minigames* designed to educate and try to eliminate the anxiety and withdrawal syndrome that generates the abstention of snuff; and (4) a messaging system to send queries to experts in smoking cessation.

### Measures and Outcomes

The primary main outcome was continuous smoking abstinence at delivery validated by carbon monoxide (CO) concentration of at least 10 parts per million at each control test [20]. The carboxymetry was carried out by trained personnel. The secondary outcome was the total days of smoking abstinence during pregnancy.

The following variables were also collected: (1) start date of the detoxification (D-day) and weeks of gestation; (2) follow-up period, time elapsed between the date of beginning of the smoking cessation until the date of completion of the follow-up; and (3) date of completion of the follow-up, delivery date.

With these variables, primary outcomes were calculated: time interval between the start of smoking cessation until relapse or end of follow-up (delivery). The follow-up period was considered ended with the following: (1) the participant decided not to continue in the study and withdrew; (2) the patient lost contact, and we had no more information on them; and (3) the completion of the follow-up on delivery.

### Ethical Considerations

Ethics approval for the study was obtained from the Ethics Committee for Clinical Research IDIAP Jordi Gol (P18/056). The information sheet to invite participants described the aim, procedures, security, and confidentiality of data and also informed about participants’ rights to refuse participation. An informed consent was collected from all participants. The study observed the current laws at the time it was conducted.

### Statistical Analysis

A descriptive analysis was performed on the results, dividing the pregnant women into 2 groups: an intervention group and a control group.

At the beginning of the study, the comparability of the test and control groups was evaluated. The means and SDs of the quantitative variables were described if they had a normal distribution, and the qualitative variables were described with percentages and CIs. The quantitative variables were compared by their means and qualitative variables using the Pearson chi-square or Fisher exact tests to assess if their presence was significantly different between the 2 groups.

Survival analyses were carried out with Cox regression model and Kaplan-Meier method, considering as an event the fact of relapsing in the smoking habit, with treatment group (intervention or usual care) entered as the main effect. The 2 groups were compared using the log-rank test. All statistical analyses were performed using SPSS version 18.0 statistical software (IBM Corp) and were 2-sided with a level of significance of alpha=.05.

## Results

### Patient Flow

After exclusion, because of no interest in participation and language barriers (n=2), 42 pregnant smoking women were recruited, and participants were distributed to either the intervention group (n=21) or the control group (n=21; Figure 2).

### Participant Characteristics

There were no significant differences in age, gender, premedication use, and previous cessation attempts between the 2 groups. The distribution of age and gender was the same across the 2 groups (Table 1).

### Smoking Cessation

#### Abstinence on Delivery

The proportion of participants who remained without smoking until delivery was significantly higher in the intervention group (57.1%, 12/21) than that in the control group (14.3%, 3/21; hazard ratio=4.31; 95% CI 1.87-9.97; *P*=.001).

**Figure 4:**
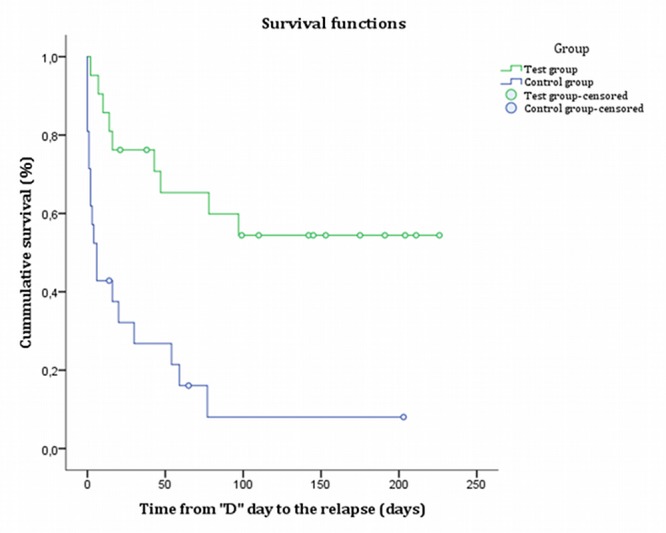
Cumulative survival curve by groups (censored: subjects with no event of smoking during the observation time).

The Kaplan-Meier survival curve (Figure 4) shows that the intervention group has a higher abstinence rate compared with the control group.

The statistical contrast log-rank, with a value of 13.91 *(P*<.001), concludes that there are statistically significant differences in cumulative survival curves between both groups.

#### Total Days without Smoking

Mantel-Haenszel method analysis comparing means of total days without smoking until delivery showed a significant difference between the intervention group (mean 139.75, SD 21.76) and the control group (mean 33.28, SD 13.27; *P*<.001).

**Table 1:** Baseline study participant characteristics.

Characteristic		Control group, n=21^a^	Intervention group, n=21^a^	*P* value
**Demographics**				
	Age (years), mean (SD)	30.43 (6.02)	31.67 (4.90)	.47
	Body mass index, mean (SD)	23.09 (5.57)	23.77 (5.08)	.68
	High school or less education, n (%)	14 (67)	16 (76)	.50
**Smoking and quitting behavior**				
	Cessation attempts, mean (SD)	0.67 (0.66)	0.95 (1.16)	.33
	Previous carboxymetries, mean (SD)	15.33 (7.91)	18.00 (7.04)	.26
	Age at which they started smoking, mean (SD)	15.43 (1.69)	14.52 (4.08)	.35
	Days from “D-day” to delivery, mean (SD)	138.81 (59.76)	152.33 (46.99)	.42
	Smoking couple, n (%)	16 (76)	17 (81)	.70
	Carbon monoxide at the beginning of study, mean (SD)	16.82 (11.19)	16.26 (8.65)	.87
	Moderate-to-high dependence, n (%)	9 (42.9)	6 (28.6)	.33
**Weekly cigarette consumption, mean (SD)**				
	Before pregnancy	20.60 (10.72)	19.12 (5.86)	.63
	When pregnant	8.57 (6.32)	7.62 (3.54)	.55
**Obstetric history**				
	Previous pregnancies, mean (SD)	1.24 (1.09)	1.00 (1.10)	.48
	Pregnancy weeks, mean (SD)	37.57 (4.85)	38.52 (1.86)	.41
	Frist trimester of pregnancy, n (%)	10 (48)	12 (57)	.54
**Type of delivery, n (%)**				
	Eutocic	13 (62)	10 (48)	.35
	Dystocic	2 (10)	3 (14)	.35
	Caesarean	5 (24)	8 (38)	.50
	Abortion	1 (5)	0	>.99

^a^One case excluded as it was lost to follow-up.

## Discussion

### Principal Findings

The findings of this pilot study suggest that the use of a mobile serious game may be an effective adjuvant intervention to contemporary management of smoking cessation in pregnant women. The results of this pilot trial showed that the intervention group had significantly higher continuous abstinence, validated by the CO carboxymeter, than the control group.

Despite other studies having shown that mobile apps are effective for quitting smoking, the Tobbstop app research on the effect of gamification as a new tool in smoking cessation has to be considered. Further research on how serious games could improve the overall satisfaction and helpfulness of these apps is needed before their implementation for medical use. More studies on serious games as Tobbstop would contribute to evaluate evidence-informed impact in smoker pregnant women willing to quit.

In addition, the reach that smoking cessation serious games, such as Tobbstop, can have, particularly for very sensitive populations such as pregnant women, supports the relevance and need for mHealth smoking cessation interventions. Smartphone ownership is nearing saturation among all population groups [10], especially in young and fertile women. As a result, health professionals need to consider the impact and the reach of these interventions as mHealth cessation interventions could potentially help to eliminate tobacco-related health disparities.

Given the potential for effective smoking cessation, serious games may warrant inclusion in the overall cessation picture for pregnant smoker’s women. Furthermore, uses of a serious game for health and health behavior change are not numerous, and it is important that studies such as this are conducted, and findings, particularly if they support the effects of these tools, need to be published.

### Limitations

There are several limitations and the most important are its sample size, its open character and the absence of usability data.

The study describes only the pregnant women participating in the trial [18] and no formal power calculation was undertaken for that sample. In other hand, pregnant women and health professionals know that they are involved in the intervention, which could create selection bias. And there is also a lack of metrics about the use or intensity of use that could be very interest to evaluate process outcomes.

The fact that the mobile app and the protocol are designed for a broader population and not specifically for pregnant smokers could obviate some specific and interesting aspect over the impact in pregnancy, so a compilation of the differences and suggestions observed should be analyzed for possible improvements in the future design according to this specific population. The lack of rewards and specific information in relation to quit smoking during pregnancy could have influenced the effective evaluation of the tool.

In addition, although the monitoring of participants with a normal follow-up gestation was guaranteed, there were some participant losses, for example, in pregnant women who abruptly ended their pregnancy or once labor had occurred at the time of follow-up. The motivations to attend the consultations once they deliver were reduced.

The study can also be limited because of the recent creation of the service in an SRHU environment that did not allow to estimate the success, types of pregnancy, and volume of participants in advance.

### Comparison With Prior Work

App stores attract millions of users seeking apps for their smartphones. Google Play (previously known as Android Market) and Apple App Store are nowadays the most widely used. In 2012, a study published in the *American Journal of Preventive Medicine* examined the content of popular apps for smoking cessation for both, iPhone, with 252 apps, and Android operating systems, with 148 apps, and the results indicate that popular apps lacked many elements recommended for quitting smoking and needed a better integration with the clinical practice guidelines and other evidence-based practices [21]. In 2017, a careful review to identify the percentage of scientifically supported apps for smoking cessation available to consumers, found that only 11 out of 158 reviewed, met inclusion criteria [22].

Some studies designed specifically with apps for pregnant smokers [23,24] have reported promising results with digital smoking cessation interventions, but despite their systematic development and usability testing, engagement was low and did not appear to increase smoking abstinence during pregnancy [25]. A recent study seems to show that choice of an app for smoking cessation can be influenced by its immediate appearance and “social successes” [26]. Design features that improve motivation, autonomy, personal relevance, and credibility can be important for commitment, and serious games could be an additional tool to do so.

There are currently no published studies with quantitative results for gamified health apps of smoking cessation. Few smoking cessation interventions with serious games have been developed, and most of them have only been evaluated in a qualitative way [10,27-29] or as a future framework [30]; yet, ample room remains to improve the evidence of their real impact on cessation.

Some studies have analyzed the use of games to help pregnant women stop smoking as part of a main app of SMS text messages [31,32], but these apps consider these trivia games only as complementary support. Recent studies with games suggest that virtual reality seems to be an effective methodology to explore cessations in young adult smokers [33], but there is not enough evidence to conclude their usefulness, as most of the literature on health games does not specify its methodologies [34].

However, to our knowledge, no study has been published on the development or evaluation of serious smoking cessation games specifically for pregnant smokers.

The demonstration of the usefulness of serious games for health as a tool to stop smoking in pregnant women warns us about the need for more analytical and experimental research that uses more exhaustive methods to recruit participants, which will be essential to confirm and expand the results of this study.

### Conclusions

In summary, the results of this pilot study introduce Tobbstop as a potentially effective and attractive adjunct therapy to existing interventions aimed at quitting tobacco use in pregnancy. Aligned with this, Tobbstop’s efficacy should be evaluated further in a bigger trial with more participants.

## Abbreviations

CO: carbon monoxide
CONSORT: Consolidated Standards of Reporting Trials
mHealth: mobile health
SMS: short message service
SRHU: sexual and reproductive health unit

## Appendix

Multimedia Appendix 1 CONSORT-EHEALTH checklist (V 1.6.1).
