# Figure Correction: Exploring Efficacy of a Serious Game (Tobbstop) for Smoking Cessation During Pregnancy: Randomized Controlled Trial

**DOI:** 10.2196/14381

**Published:** 2019-07-11

**Authors:** Francesc X Marin-Gomez, Rocio Garcia-Moreno Marchán, Anabel Mayos-Fernandez, Gemma Flores-Mateo, Esther Granado-Font, Maria Luisa Barrera Uriarte, Jordi Duch, Cristina Rey-Reñones

**Affiliations:** 1 Servei d'Atenció Primària d'Osona Gerència Territorial de la Catalunya Central Institut Català de la Salut Vic Spain; 2 Unitat de Suport a la Recerca Catalunya Central Institut Universitari d'Investigació en Atenció Primària Jordi Gol Sant Fruitós de Bages Spain; 3 Health Promotion in Rural Areas Research Group Institut Català de la Salut Sant Fruitós de Bages Spain; 4 Digital Care Research Group Universitat de Vic–Universitat Central de Catalunya Centre for Health and Social Care Research Vic Spain; 5 Sexual and Reproductive Health Unit Servei d'Atenció Primària d'Osona Institut Català de la Salut Vic Spain; 6 Grup de Recerca en Tecnologies de la Informació en Atenció Primaria Unitat de Suport a la Recerca Tarragona-Reus Institut Universitari d'Investigació en Atenció Primària Jordi Gol Reus Spain; 7 Unitat d’Anàlisi i Qualitat Xarxa Sanitària i Social Santa Tecla Tarragona Spain; 8 Departament d'Infermeria Facultat d'Infermeria Universitat Rovira i Virgili Tarragona Spain; 9 Centre d'Atenció Primària Horts de Miró (Reus-4) Gerència d'Àmbit d'Atenció Primària Camp de Tarragona Institut Català de la Salut Tarragona Spain; 10 Centre d’Atenció Primària La Granja (Tarragona-2) Gerència d’Àmbit d’Atenció Primària Camp de Tarragona Institut Català de la Salut Torreforta,Tarragona Spain; 11 Departament d'Enginyeria Informàtica i Matemàtiques Universitat Rovira i Virgili Tarragona Spain

The manuscript “Exploring Efficacy of a Serious Game (Tobbstop) for Smoking Cessation During Pregnancy: Randomized Controlled Trial” (JMIR Serious Games 2019;7(1):e12835) was erroneously published with a duplicate of Figure 2 in place of Figure 3 due to a display error on the publisher’s website. The correct version of Figure 3 can be seen below, and now also appears in the appropriate place in the corrected manuscript.

The correction will appear in the online version of the paper on the JMIR website on July 11, 2019, together with the publication of this correction notice. Because this was made after submission to PubMed, PubMed Central, and other full-text repositories, the corrected article also has been resubmitted to those repositories.

**Figure 3:**
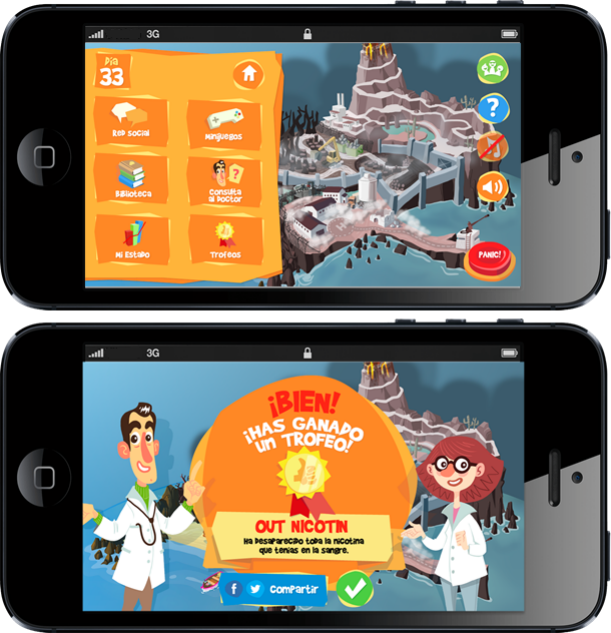
Screenshots of Tobbstop.

